# First-Principles Studies for Electronic Structure and Optical Properties of *p*-Type Calcium Doped α-Ga_2_O_3_

**DOI:** 10.3390/ma14030604

**Published:** 2021-01-28

**Authors:** Abhay Kumar Mondal, Mohd Ambri Mohamed, Loh Kean Ping, Mohamad Fariz Mohamad Taib, Mohd Hazrie Samat, Muhammad Aniq Shazni Mohammad Haniff, Raihana Bahru

**Affiliations:** 1 Institute of Microengineering and Nanoelectronics (IMEN), Universiti Kebangsaan Malaysia (UKM), Bangi 43600, Selangor, Malaysia; abhay.nano17@gmail.com (A.K.M.); p103776@siswa.ukm.edu.my (L.K.P.); aniqshazni@ukm.edu.my (M.A.S.M.H.); raihanabahru@ukm.edu.my (R.B.); 2 Faculty of Applied Sciences, Universiti Teknologi MARA (UiTM), Shah Alam 40450, Selangor, Malaysia; mfariz@uitm.edu.my; 3 Ionic Materials & Devices (iMADE) Research Laboratory, Institute of Science, Universiti Teknologi MARA (UiTM), Shah Alam 40450, Selangor, Malaysia; mohdhazrie@uitm.edu.my

**Keywords:** first-principles, density functional theory, pure α-Ga_2_O_3_, Ca-doped α-Ga_2_O_3_, electronic structure, optical properties

## Abstract

Gallium oxide (Ga_2_O_3_) is a promising wide-band-gap semiconductor material for UV optical detectors and high-power transistor applications. The fabrication of *p*-type Ga_2_O_3_ is a key problem that hinders its potential for realistic power applications. In this paper, pure α-Ga_2_O_3_ and Ca-doped α-Ga_2_O_3_ band structure, the density of states, charge density distribution, and optical properties were determined by a first-principles generalized gradient approximation plane-wave pseudopotential method based on density functional theory. It was found that calcium (Ca) doping decreases the bandgap by introducing deep acceptor energy levels as the intermediate band above the valence band maximum. This intermediate valence band mainly consists of Ca 3p and O 2p orbitals and is adequately high in energy to provide an opportunity for *p*-type conductivity. Moreover, Ca doping enhances the absorptivity and reflectivity become low in the visible region. Aside, transparency decreases compared to the pure material. The optical properties were studied and clarified by electrons-photons interband transitions along with the complex dielectric function’s imaginary function.

## 1. Introduction

Gallium oxides (Ga_2_O_3_) are entitled to five different polymorphs, namely α, β, γ, ε, and δ. Among them, corundum structure α-Ga_2_O_3_ has emerged as a next-generation power semiconductor material for a sensor, solar-blind UV-photodetectors, photocatalyst, and high-power devices [1,2,3,4]. The ultra-wide bandgap semiconductor α-Ga_2_O_3_ enables high temperature and high voltage device operation such as field-effect transistor (FET) and Schottky barrier diode (SBD). α-Ga_2_O_3_ is privillaged of superior properties such as a smaller electron effective mass, higher breakdown field, and larger Baliga figure of merit [5,6,7] which have capabilities to go beyond existing technologies to 4H–SiC (3.26 eV) [8] and GaN (3.44 eV) in the power industry [9]. 

Band structure engineering would open new avenues in device applications [10]. Doping with particular elements in practical applications can boost the material’s electrical and optical properties and maximize the devices’ efficiency. Mostly, α-Ga_2_O_3_ is privileged with good *n*-type doping conductivity due to its deep donor oxygen defect nature [11]. Silicon (Si) and Tin (Sn) are highly studied *n*-type shallow donors with a small ionization energy [11,12]. In contrast, high-quality *p*-type α-Ga_2_O_3_ is challenging due to the lack of hole conductivity, low solubility, and activation rate of dopants which largely impede the formation of the *p–n* junction bipolar semiconductor devices [13,14,15,16]. Despite several experimental reports on α-Ga_2_O_3_, its theoretical analysis is relatively small and started to receive attention recently. The theoretically predicted Gr-I and Gr-II metals shows *p*-type nature by doping on Ga_2_O_3_ as reported by Tang et al. [17]. In 2019, Dong et al. investigated the magnesium (Mg) element substitute at the gallium site that had proven to be a good *p*-type conductivity in α-Ga_2_O_3_ through first-principles studies [18]. Tang et al. reported that the same alkaline earth metal group, calcium (Ca), plays a deep acceptor element and studied the electronic structures and optical properties of *p*-type Ca doped on a β-Ga_2_O_3_ first-principle calculation [17]. There is a lack of in-depth analysis and systematic study of new strong acceptor doping studies for the bipolar *p-n* junction of electronic and optical α-Ga_2_O_3_ devices, which is of high demand for further exploration of potential α-Ga_2_O_3_ films. 

In this research, Gr-IIA alkaline metal Ca doping α-Ga_2_O_3_ had reported the electronic structure and optical properties by the first-principles studies based on density functional theory (DFT). The work aims to find Ca potential by substituting the Ga site and supporting this theoretical investigation for future experimental work. Ca attributes of small ionization energy quickly release holes and show *p*-type conductivity nature. The doping of Ca contributes to a slight reduction in the optical bandgap due to the addition of the impurity band into the valance band (VB). The optical absorption spectra have shown a redshift of the absorption edge towards the visible-infrared region.

## 2. Computational Details

In this study, all the calculations were performed by using the Cambridge Serial Total Energy Package (CASTEP) code in Materials Studio (MS) 6.1 software based on the DFT [19]. The ultra-soft pseudopotential approach is used for general energy. The electronic wave function is unfolded with plane wave base groups, and ion potential is replaced by ultra-soft pseudopotential [20]. Ultrasoft pseudopotentials technology interacts with ions and electrons with total energy convergence 5 × 10^−6^ eV/atom [21]. Cut-off plane-wave energy is set at 380 eV for total-energy, band structure, and spectra calculations. It has been shown that this functional exchange-correlation gives rise to wide-gap semiconductor atomic geometries with strong ionic bonds in excellent accordance with measured values. The exchange-correlation potential is defined by the generalized gradient approximation (GGA) in the Perdew-Burke-Ernzerhof (PBE) function and the local density approximation (LDA) [22]. The LDA utilization in this simulation work was used to compare the parameters, cell volume, and cell angle from the feature of the GGA-PBE. The electronic and optical characterization is then determined based on the structural geometry optimization to make the calculations effective and trackable [18]. The optimization of all calculated structures was done until the Hellmann Feynman force became less than 0.01 eV/Å (1 Å = 0.1 nm). Broyden-Fletcher-Goldfarb Shanno (BFGS) is a reliable approach to optimize the structure by full relaxation of the lattice constant and internal coordinates [23]. For maximum stress and displacement, the convergence criterion was 0.02 GPa and 5 × 10^−4^ Å, respectively. A primitive cell composed of 30 atoms (12 Ga atoms and 18 O atoms) was used to study the electronic structure and optical properties of the Ca doped α-Ga_2_O_3_. A 3 × 3 × 2 k-point mesh of Monkhorst–Pack methods was used to integrate the Brillouin zone [24]. Ga, O, and Ca valence shell electrons configuration were [Ar] 3d^10^4s^2^4p^1^, [He] 2s²2p⁴, and [Ar] 4s², respectively.

## 3. Results and Discussion

### 3.1. Geometrical Structures

**Figure 1:**
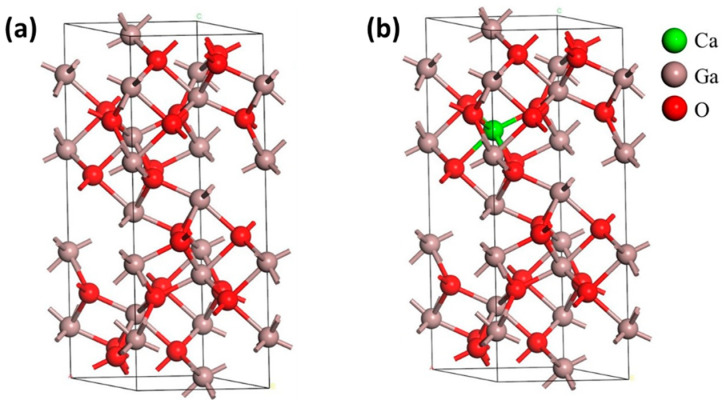
The crystal structure of the primitive cell (**a**) intrinsic α-Ga_2_O_3_ and (**b**) Ca-doped α-Ga_2_O_3_. Ga, O, and Ca atoms are denoted by brown, red, and green colors, respectively.

Firstly, geometry optimization was implemented using the BFGS method [25]. The most stable structure was achieved after the fully relaxed cell parameter and volume by reducing the energy state [20]. The investigated optimized crystal structure of intrinsic α-Ga_2_O_3_ and Ca-doped α-Ga_2_O_3_ is shown in Figure 1a,b. The lattice parameters a, b, c, and unit cell volume V are increased after Ca doping due to the larger atomic radius of Ca than that of Ga. Meanwhile, the bandgap E_g_ decreases with Ca doping.

Figure 1 shows the typical corundum structure of α-Ga_2_O_3_ with R$\bar{3}$c symmetry, and the crystalline cell comprises six Ga_2_O_3_ formula units. In the unit cell of α-Ga_2_O_3,_ oxygen anions are almost closely hexagonally packed, and gallium atoms occupy octahedral two-third sites. Every gallium octahedron shares one face and three edges with the other three octahedra sites. The gallium octahedral sites are distorted by the crystal lattice [7]. We investigated and explored the electronic and optical properties of Ca doped α-Ga_2_O_3_ by substitution of Ga atoms lowest energy preferred sites. Table 1 lists all the optimized lattice parameters, and cell volume of pure and Ca doped α-Ga_2_O_3_ material as compared to experimental results (a = b = 4.983, c = 13.433) [26]. The experimental value for α-Ga_2_O_3_ has a lattice parameter and cell volume closer to that of the LDA calculation. In addition, the error is less than 1.5%, ensuring the reliability of our results. It has been shown that the calculated LDA is more precise and accurate than GGA-PBE according to the experimental reports on the lattice parameter of hexagonally structured α-Ga_2_O_3_. Therefore, in this study, the electronic structure and optical properties of Ca doped α-Ga_2_O_3_ were calculated using the DFT-LDA approach.

The bond lengths for pure and Ca-doped α-Ga_2_O_3_ are shown in Table 2. It is shown that the atomic radius of the Ga atom is smaller than that of the Ca atom according to the periodic table. Therefore, the Ga–O bond length is shorter than the Ca–O and O–O bonds in Ca-doped α-Ga_2_O_3_. This situation is the same as the pure α-Ga_2_O_3,_ where the Ga–O bond length is shorter than O–O bonds. After Ca doping, the overall bond length is larger than before doping due to the concentration of Ca^2+^ ions.

### 3.2. Electronic Structure

#### 3.2.1. Electron Charge Density

**Figure 2:**
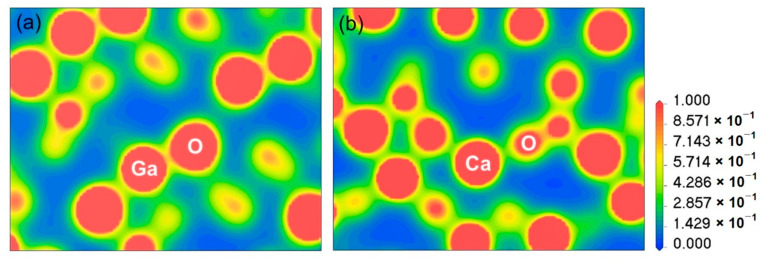
Distribution of electron density of (**a**) pure α-Ga_2_O_3_ and (**b**) Ca-doped α-Ga_2_O_3._

The ionic and covalent bonding-based charge density contours of intrinsic α-Ga_2_O_3_ and Ca-doped α-Ga_2_O_3_ are shown in Figure 2a,b, respectively. The charge density distributions can be used to assess crystal bonding characteristics. In α-Ga_2_O_3_, the ionic or covalent structures are a contentious subject and have vital facts to understand the charge transport property [23,24]. The nuclei are bound by the charge density, which is shared between them. It has been shown that certain covalent bonding characters can exist between Ga and O atoms [24]. The electrons are located around the O atoms in the hexagonal phases, and there is no region of bond localization, indicating a predominantly ionic bond.

In Figure 2b, a major reflection is observed in the distribution of charge density between the doping Ca atom and native atoms. By incorporating Ca, the oxygen atom’s nearest electrons population reduce compared to distant oxygen atoms, as presented in Figure 2b. Consequently, only the closest few electrons of the O atom donate to the Ca atom, leaving holes in the O atom to be *p*-type. The Ca atom loses all valence electrons while the electron density around the O atoms increases evidently and further decreases in the region between the Ca atom and the nearest O atom. A strong ionic bonding appears between Ca and the nearest neighbor O atoms. Meanwhile, Ca-doped α-Ga_2_O_3_ gives the outer shell electrons 4s^2^ and partial 3p^6^ electrons of Ca atoms coupled with O 2p electrons.

#### 3.2.2. Band Structure and Density of States

**Figure 3:**
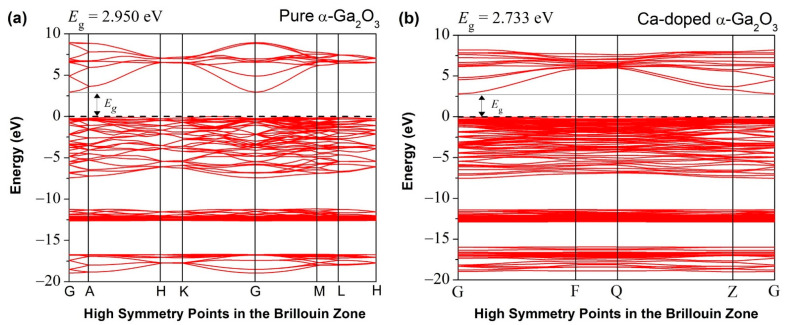
Energy band structure diagram of (**a**) pure α-Ga_2_O_3_ and (**b**) Ca-doped α-Ga_2_O_3_; Fermi level is set to zero.

**Figure 4:**
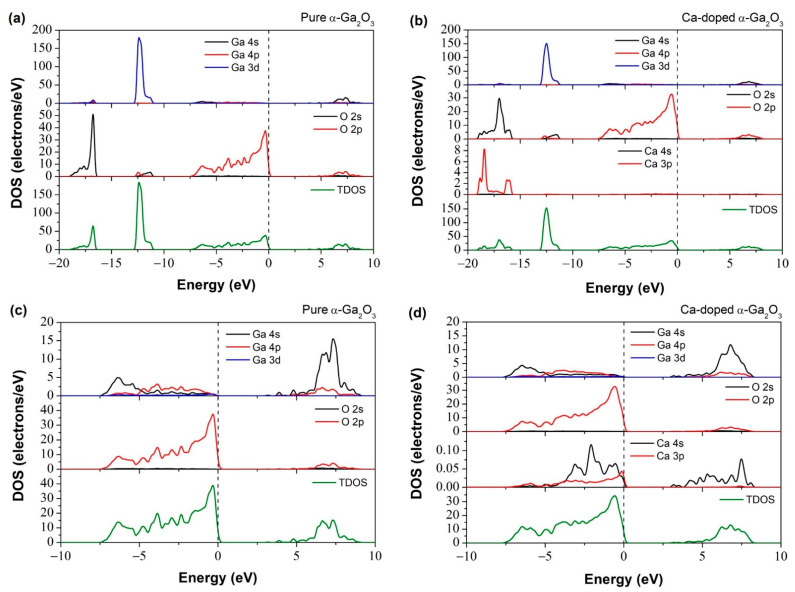
The density of states of (**a**) pure α-Ga_2_O_3_, (**b**) Ca-doped α-Ga_2_O_3_, (**c**) pure α-Ga_2_O_3_ (−10 to 10 energy window), and (**d**) Ca-doped α-Ga_2_O_3_ (−10 to 10 energy window).

The first-principal calculations of the electronic band structure of undoped α-Ga_2_O_3_ are shown in Figure 3a. The density of states is shown in Figure 4a,c at different energy windows. The bandgap energy is usually determined between the valence band maximum (VBM) and the conduction band minimum (CBM), placed at the Brillion region’s G point. The Fermi level is situated at 0 eV of the energy scale. The large disparity of theoretically calculated DFT-LDA bandgap (2.950 eV) and experimental band gap (5.3 eV) is due to the ground state DFT results of strong Coulomb correlation and exchange-correlation potential between the excited electrons in the underestimated LDA bandgap [27].

The electronic band structure of pure α-Ga_2_O_3_, as shown in Figure 3a, is represented by the crystal structure made with the 12 Ga atoms and 18 O atoms of primitive cells. Figure 4a shows the uppermost valence band is a major contribution of O 2p states with a width from about −0.12 to −7.43 eV and a minor contribution of Ga 3d, 4p, 4s, and O 2s orbitals which are located between −11.5 and −18.5 eV. CBM consists of Ga 4s orbital states, and lies between 2.32 to 8.46 eV [26]. The valence band’s edge is reasonably flat, and the effective mass at VBM is relatively heavy. Characteristics of CBM is almost equally dispersed everywhere.

The electronic band structure of Ca-doped α-Ga_2_O_3_ is shown in Figure 3b, and its corresponding DOS is shown in Figure 4b,d, at different energy values. The acceptor dopant has been introduced above the valence band and intersected with the Fermi level. The Ca-α-Ga_2_O_3_ has a direct bandgap with the most upper part of the valence band and the lowest part of the G point’s conduction band. It is noticed that the DFT-LDA calculated bandgap of Ca-doped α-Ga_2_O_3_ is 2.733 eV which is 0.217 eV less than that of pure α-Ga_2_O_3_. In Ca-doped systems, the Ca 3p state contributes to the VBM, and the CBM is contributed to by Ga-4s and Ca-3p states. Ca creates dopant states near the topmost of VBs and shift the valence band edge at higher energy and reduce the bandgap.

**Figure 5:**
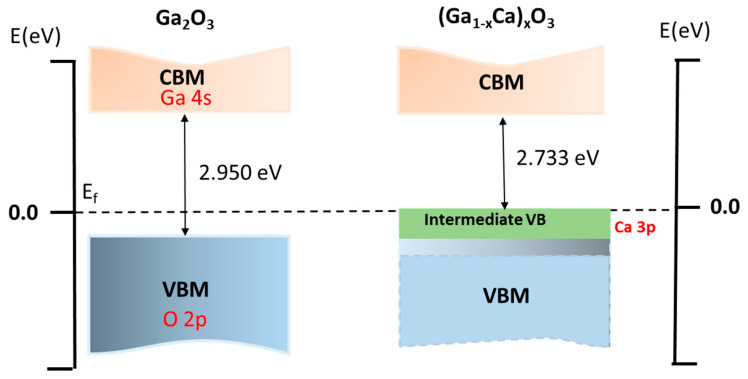
Schematic representation of the effects of adding Ca on the electronic band structure of α-Ga_2_O_3_. (Ga_1−x_Ca_x_)_2_O_3_ with x = 1/30. All energies are in eV. The bands showed in green represent the intermediate valence band, composed of hybridized Ca 3p and O 2p orbitals.

The Ca 4s state shows very narrow bands at −15.79 to −19.02 in Figure 4b. The impurity levels lead to the excess holes over the upper part of the valence band [27]. The Ca doped cases’ highest occupy degree shifts dramatically downward by 0.12 eV due to the far higher energy of Ca 4s compared to Ga 3s orbitals. At around −16.1 to −18.6 eV, six bands are mainly composed of O 2s states and at 2.07–6.94 eV, six bands are mainly composed of O 2p states (Figure 4b). At around −11.2 to −12.9 eV, 20 bands consist mainly of Ga 3d states.

(Ga_1−x_Ca_x_)_2_O_3_ alloys bandgap energy with the incorporation of Ca impurity is schematically shown in Figure 5. In α-Ca_2_O_3_, 3.33% of Ca concentrations were produced by replacing one Ga atom with Ca, respectively. Ca substitutes Ga octahedral sites and fully occupied band energy higher than the original valence band of the Ga_2_O_3_. This intermediate band originates from the coupling of Ca 3p, which lies below the O 2p band that forms the higher-lying valence bands, as shown in Figure 5. Furthermore, the intermediate valence band shows that a downward movement of the CBM was formed, attributed to the attraction between the Ca intermediate valence band and the CBM of the host. This situation resulted in a decrease in the bandgap of the (Ga_1−x_Ca_x_)_2_O_3_ alloy with the doping of Ca.

### 3.3. Optical Properties

#### 3.3.1. Theoretical Description and Optical Properties

The optical properties of pure α-Ga_2_O_3_ and Ca doped α-Ga_2_O_3_ can be studied based on the dielectric function, absorption coefficient, and reflectivity. The optical transitions are related to inter-band and intra-band transitions in the crystal structure of solid materials. The inter-band transitions mainly contributed to the semiconductor rather than intra-band transitions to semiconducting crystal structure. The dielectric function *ε*(*ω*) has a frequency dependence linear electromagnetic interaction response of incident photons with electrons and is separated into the real part $\varepsilon_{1}$(*ω*) and imaginary part i$\varepsilon_{2}$(*ω*). It is difficult to achieve accurate optical constants due to the underestimation of the bandgap. Hence, the optical property results were corrected by scissors operators according to experimental results. Scissors operators = 5.3 eV−2.950 eV = 2.35 eV, where 2.950 is theoritical DFT-LDA calculated bandgap and 5.3 eV is experimental bandgap [28]. The scissors operators used to shift all conduction levels to match the measured value of the bandgap. All-optical properties can be described from the complex dielectric constant in Equation (1) [29]:

(1) $$\varepsilon \left(\omega \right)=\varepsilon_{1}\left(\omega \right)+i\varepsilon_{2}\left(\omega \right)$$

The imaginary dielectric part $\varepsilon_{2}$(*ω*) semiconductors are an essential parameter for determining optical properties for different designs of optoelectronic devices. The dielectric function imaginary part $\varepsilon_{2}$(*ω*) is associated with the dielectric loss of energy or absorption of light described by the absorption coefficient in the material while the real part $\varepsilon_{1}$(*ω*) is associated with the stored energy within the material (degree of polarization). The complex dielectric function imaginary part $\varepsilon_{2}$(*ω*) is determined by summing the transitions between occupied and unoccupied electronic states, as reported in Equation (2) [22]:

(2) $$\varepsilon_{2}\left(\omega \right)=\left(\frac{4\pi^{2}e^{2}}{m\omega^{2}}\right)\sum_{i,j}\int \langle i|M|j\rangle^{2}f_{i}\left(1-f_{i}\right)\;\times \;\delta \left(E_{jk}-E_{ik}-\omega \right)d^{3}k$$

where m is the mass of free electrons, e is the electron charge, M is the dipole matrix, and ω is the frequency of incident photons. i and j are the initial and final states, respectively, f_i_ denotes the Fermi distribution function and i-th state with wave function vector k. The dielectric function real part $\varepsilon_{1}$(ω) can be derived from the imaginary part i$\varepsilon_{2}$(*ω*) by the Kramers–Kronig dispersion Equation (3) [30]:

(3) $$\varepsilon_{1}\left(\omega \right)=1+\frac{2}{\pi }P\int_{0}^{ᴽ}\frac{\omega \varepsilon \left(ὡ\right)d\omega }{\left({ὡ}^{2}-\omega^{2}\right)}$$

where P is the principal value of the integral. The absorption coefficient can be derived from the dielectric function as shown in Equation (4) [31]:

(4) $$\alpha \left(\omega \right)=\sqrt{2}(\omega )[\sqrt{\varepsilon_{1}{\left(\omega \right)}^{2}+\varepsilon_{2}{\left(\omega \right)}^{2}}-\varepsilon_{1}\left(\omega \right){]}^{1/2}$$

#### 3.3.2. Dielectric Function

The dielectric constant is directly proportional to the crystal’s polarizability, representing the deformability of the electronic distribution, and relates the shape of the valence band charge density. Oxygen ions are supposed to contribute to polarizability in such a highly ionic substance as Ga_2_O_3_. As was predicted in the current study, a difference of dielectric properties between Ca and α-Ga_2_O_3_ is projected. A considerably larger dielectric constant of α-Ga_2_O_3_ compared to Ca doped α-Ga_2_O_3_ can be drawn to a lightly dense ion package and a large number of Ga atoms coordinated in the α-Ga_2_O_3_ lattice [7].

**Figure 6:**
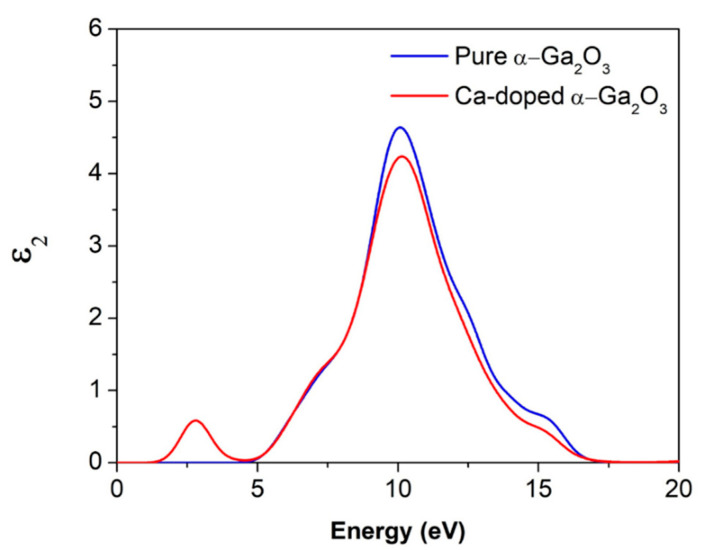
The imaginary part of the complex dielectric function for intrinsic α-Ga_2_O_3_ and Ca doped α-Ga_2_O_3_.

From Figure 6, the calculated imaginary dielectric function of intrinsic α-Ga_2_O_3_ and Ca-α-Ga_2_O_3_ were plotted and compared. The main peaks are 10.12 eV of pure and Ca-doped α-Ga_2_O_3_, respectively, corresponding to the electrons’ transitions from the upper state O-2p valence band to the lower state Ga-4s conduction band. The Ca-doped α-Ga_2_O_3_ peak is smaller than that of intrinsic α-Ga_2_O_3_ and is enhanced towards the lower energy site. Moreover, this Ca doping absorption peak at 2.9 eV indicates that the absorption spectra increase from the UV to the visible region, and the average optical transmittance decreases.

#### 3.3.3. Absorption and Reflectivity

**Figure 7:**
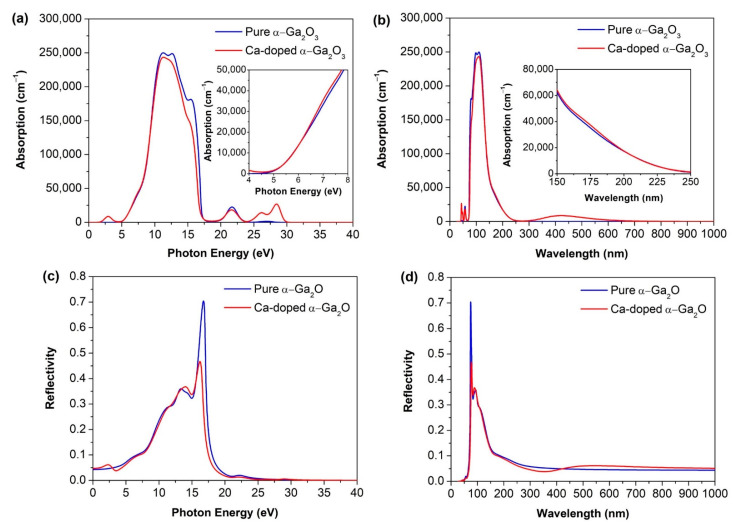
The absorption spectrum of (**a**) intrinsic α-Ga_2_O_3_ and Ca-doped α-Ga_2_O_3_ in energy, (**b**) intrinsic α-Ga_2_O_3_ and Ca-doped α-Ga_2_O_3_ in wavelength, the reflectivity of (**c**) intrinsic α-Ga_2_O_3_ and Ca-doped α-Ga_2_O_3_ in energy, and (**d**) intrinsic α-Ga_2_O_3_ and Ca-doped α-Ga_2_O_3_ in wavelength window regime.

The absorption spectra of intrinsic α-Ga_2_O_3_ and Ca-doped α-Ga_2_O_3_ in the range of energy (0 to 40 eV) and wavelength (0–1000 nm) are shown in Figure 7a,b. The presence of a high energy peak at 11.5 eV into the deep ultraviolet region is shown in both cases of α-Ga_2_O_3_ and Ca-doped α-Ga_2_O_3_. These peaks are associated with the inter-band transition from the valance band O 2p occupied states to the conduction band Ga 4s unoccupied states. From Figure 7a, it is worth noticing that the absorptivity of α-Ga_2_O_3_ is in the deep UV spectrum due to its semi-insulating nature. Meanwhile, Ca-doped α-Ga_2_O_3_ appears as a sharp new small peak at 2.9 eV which originated from the inter-band transitions of C 3p from the VBM to these CBM that promote $\varepsilon_{2}$ and enhanced absorption in the visible region.

Figure 7a,b show that the absorption spectra have a redshift with the Ca-doping. This shifting is mainly because of the hole carriers generated after the Ca doping so that the Fermi level enters the valence band, which is realized in the density of the states. The same phenomenon was observed in the absorption spectrum wavelength window in both cases of undoped and Ca doped α-Ga_2_O_3_ (Figure 7b). As shown in Figure 7b, the absorption spectra indicate that the transparency decreases with Ca doping due to enhancement of absorbance in the visible region.

Figure 7c,d show the reflection spectra of intrinsic α-Ga_2_O_3_ and Ca-doped α-Ga_2_O_3_ in the energy and wavelength window regime. For pure α-Ga_2_O_3_, a sharp main intense peak appears at 16 eV in the energy range and 70.45 nm in the wavelength range, and it decreases from the deep UV-visible to infrared region. Meanwhile, for the Ca doped α-Ga_2_O_3_ system, two major peaks appear at the same position as the pure phase, but reflectivity is lower than that of the pure one. These characters drag material potential towards optoelectronics and bipolar device application.

## 4. Conclusions

The substitutional Ca doping on the α-Ga_2_O_3_ electronic, structural, and optical properties was studied through a first-principles calculation based on the DFT. The Ca doping predominantly affects the band structure and optical properties of pure α-Ga_2_O_3_. The DFT-LDA calculated bandgap of α-Ga_2_O_3_ is decreased to 2.950 eV after Ca doping and the forbidden bandwidth appeared at the top of the valance band. The results obtained show that Ca-doped α-Ga_2_O_3_ is an obvious variation of energy band structures and the charge density distribution. The acceptor energy states are composed of Ca 3p and O 2p orbital states. The Ca-doped α-Ga_2_O_3_ dynamic dielectric function is observed. The most important absorption peak shifted in the visible regime; it realizes *p*-type α-Ga_2_O_3_ but not a strong *p*-type candidate for optoelectronic device application. However, the lack of experimental results about the optical properties of Ca doped α-Ga_2_O_3_ requires further experimental works to compare with our simulation results.

## Figures and Tables

**Table 1:** The lattice parameters (a, b, and c) and unit cell volume (V) of basic α-Ga_2_O_3_ and Ca doped α-Ga_2_O_3_ crystal structure. The values were compared with theoretical and experimental results.

Phase		a (Å)	b (Å)	c (Å)	V (Å^3^)
**Pure Ga_2_O_3_**	LDA-CAPZ	4.965 (−0.36%)	4.965 (−0.36%)	13.338 (−0.71%)	284.747 (−1.42%)
	GGA-PBE	5.074 (1.83%)	5.074 (1.83%)	13.664 (1.72%)	304.656 (5.47%)
	GGA-PBEsol	5.028 (0.90%)	5.028 (0.90%)	13.511 (0.58%)	295.807 (2.41%)
	GGA-PBE (CASTEP) [6]	5.07	5.07	13.67	304.309
	Expt. [26]	4.983	4.983	13.433	288.859
**Ca-Doped Ga_2_O_3_**	LDA-CAPZ	5.010 (0.54%)	5.010 (0.54%)	13.520 (0.647%)	293.889 (1.74%)

**Table 2:** The calculated bond length of undoped and Ca-doped α-Ga_2_O_3_.

Type of Bond Length	Pure α-Ga_2_O_3_	Ca-doped α-Ga_2_O_3_
Ga–O (Å)	1.989	1.993
O–O (Å)	2.762	2.741
Ca–O (Å)	–	2.253

## Data Availability

No new data were created or analyzed in this study. Data sharing is not applicable to this article.
